# The Role of Cardioprotection in Cancer Therapy Cardiotoxicity: *JACC: CardioOncology* State-of-the-Art Review

**DOI:** 10.1016/j.jaccao.2022.01.101

**Published:** 2022-03-15

**Authors:** Torbjørn Omland, Siri Lagethon Heck, Geeta Gulati

**Affiliations:** a Department of Cardiology, Division of Medicine, Akershus University Hospital, Lørenskog, Norway; b Institute of Clinical Medicine, Faculty of Medicine, University of Oslo, Oslo, Norway; c Department of Diagnostic Imaging, Akershus University Hospital, Lørenskog, Norway; d Division of Research and Innovation, Akershus University Hospital, Lørenskog, Norway; e Department of Cardiology, Division of Medicine, Oslo University Hospital, Ullevål, Oslo, Norway

**Keywords:** anthracycline, cardiomyopathy, HER2 therapy, prevention, ACE, angiotensin-converting enzyme, ADT, androgen deprivation therapy, ARB, angiotensin receptor blocker, CMR, cardiovascular magnetic resonance, CTRCD, cancer therapy–related cardiac dysfunction, GLS, global longitudinal strain, GnRH, gonadotropin-releasing hormone, HER2, human epidermal growth factor receptor 2, LV, left ventricular, LVEF, left ventricular ejection fraction, MRA, mineralocorticoid receptor antagonist, RR, risk ratio

## Abstract

Cardiotoxicity is a relatively frequent and potentially serious side effect of traditional and targeted cancer therapies. Both general measures and specific pharmacologic cardioprotective interventions as well as imaging- and biomarker-based surveillance strategies to identify patients at high risk have been tested in randomized controlled trials to prevent or attenuate cancer therapy–related cardiotoxic effects. Although meta-analyses including early trials suggest an overall beneficial effect, there is substantial heterogeneity in results. Recent randomized controlled trials of neurohormonal inhibitors in patients receiving anthracyclines and/or human epidermal growth factor receptor 2–targeted therapies have shown a lower rate of cancer therapy–related cardiac dysfunction than previously reported and a modest or no sustained effect of the interventions. Data on preventive cardioprotective strategies for novel cancer drugs are lacking. Larger, prospective multicenter randomized clinical trials testing traditional and novel interventions are required to more accurately define the benefit of different cardioprotective strategies and to refine risk prediction and identify patients who are likely to benefit.

## Highlights

• Traditional and targeted cancer therapies are associated with risk of cardiotoxicity.
• There is incomplete evidence of the effect of cardioprotective pharmacotherapy.
• Treatment of reversible cardiovascular risk factors is highly recommended.
• Close collaboration between oncologists and cardiologists is essential.

Advances in cancer therapy, including the development of targeted therapies, have been associated with improved cancer outcomes. The resulting increase in the number of long-term survivors has led to increased attention to the potential acute and chronic side effects that may reduce the quality of life, and in some instances the life expectancy, of cancer survivors. Cardiovascular disease is considered one of the more frequent and potentially serious cancer therapy related side effects. These observations have generated increasing interest in the potential prevention and treatment of cancer therapy cardiotoxicity by both general and specific cardioprotective strategies and form the basis for the rapidly evolving field of cardio-oncology.

The development and growing use of cancer therapies that block the growth and spread of cancer by interfering with specific molecules have also characterized medical oncology during the past 20 years. Targeted therapies include human epidermal growth factor receptor 2 (HER2)–targeted therapies, tyrosine kinase inhibitors, immune checkpoint inhibitors, proteasome inhibitors, and androgen deprivation therapy (ADT). The use of targeted therapies has markedly improved cancer outcomes, including progression-free and overall survival, but their implementation has also revealed the occurrence of systemic and cardiovascular off-target toxicities. The development of left ventricular (LV) dysfunction and heart failure are common side effects of several targeted therapies, but the modes of actions and mechanisms of cardiotoxicity of various therapies differ. Targeted therapies have also been associated with increased incidence of other cardiovascular abnormalities, including myocarditis and arterial hypertension.^1^

The term *cardiotoxicity* is not uniformly or universally defined. According to the National Cancer Institute, cardiotoxicity is broadly defined as “toxicity that affects the heart.”^2^ The term may thus include toxicity not only to the myocardium but also the pericardium, endocardium, and coronary vasculature. However, the term has commonly been used in a narrower sense to mean a reduction in LV systolic function and/or the development of clinical heart failure, on the basis of the classical observations that anthracycline therapy, in a dose-dependent fashion, is associated with the risk for developing an irreversible cardiotoxic cardiomyopathy.^3^ Accordingly, the term *cardiotoxicity* has often been used interchangeably with the more recent term *cancer therapy–related cardiac dysfunction* (CTRCD).

In this state-of-the-art review, our emphasis is on the role different cardioprotective strategies play in mitigating the cardiotoxic effects of both conventional and targeted cancer therapies in adults. We include a review of the available evidence with an emphasis on recently published randomized controlled trials (Tables 1 and 2) and meta-analyses, but we also provide an overview of the objectives of important ongoing randomized trials (Table 3). Finally, we briefly summarize some practical guidance for clinicians treating patients receiving conventional and targeted anticancer therapies (Table 4).

**Table 1:** Recent Randomized Controlled Trials on Cardioprotection During Anthracycline Therapy

Trial	Trial Design	Trial Intervention	Imaging Method	N	Result of Primary Endpoint and Follow-Up Results	Result of Key Secondary Endpoints and Follow-Up Results
Pharmacologic intervention						
PRADA^22^, ^23^, ^24^	Randomized Placebo-controlled Double-blind 2 × 2 factorial	Metoprolol Candesartan Metoprolol plus candesartan Placebo	CMR	130	Primary trial: candesartan attenuated the reduction in LVEF Follow-up: no difference in change in LVEF from baseline to extended follow-up in either treatment arm	Primary trial: metoprolol attenuated the rise in troponins Follow-up: no difference in change in troponins from baseline to extended follow-up in either treatment arm
CECCY^25^^,^^26^	Randomized Placebo-controlled Double-blind	Carvedilol Placebo	Echocardiography	200	Primary trial: no effect on reduction of LVEF ≥10% from baseline Follow-up: no difference in change of LVEF at 2-y follow-up	Primary trial: carvedilol attenuated the rise in troponin I
SAFE^27^	Randomized Placebo-controlled Double-blind	Bisoprolol/enalapril/bisoprolol plus enalapril/placebo	Echocardiography (3D)	174	Bisoprolol, enalapril, and bisoprolol plus enalapril attenuated the reduction in LVEF Bisoprolol and enalapril prevented worsening in peak GLS	
Risk-guided strategy						
ICOS-ONE^33^^,^^34^	Randomized Open-label Multicenter	Enalapril Preventive treatment vs Troponin-triggered treatment	Echocardiography	273	Primary trial: no between-group differences in the incidence of troponin elevation Follow-up: no increased concentrations of cardiac troponin I at 3-y follow-up	Primary trial: no between-group differences in the incidence of CTRCD, defined as a reduction in LVEF of ≥10% to a value <50% Follow-up: no new cases of incident CTRCD at 3-y follow-up
SUCCOUR^35^	Randomized Open-label Multicenter	Surveillance with serial measurements of LVEF or with measurement of peak GLS	Echocardiography	331	No between-group difference in change in LVEF at 1-y	Use of neurohormonal therapy was significantly higher in the GLS-guided than in the LVEF-guided trial arm

3D = 3-dimensional; CECCY = Carvedilol for Prevention of Chemotherapy-Related Cardiotoxicity; CMR = cardiovascular magnetic resonance; CTRCD = cancer therapy–related cardiac dysfunction; GLS = global longitudinal strain; ICOS-ONE = International CardioOncology Society-one; LVEF = left ventricular ejection fraction; PRADA = Prevention of Cardiac Dysfunction During Adjuvant Breast Cancer Therapy; SAFE = Cardiotoxicity Prevention in Breast Cancer Patients Treated With Anthracyclines and/or Trastuzumab; SUCCOUR = Strain Surveillance of Chemotherapy for Improving Cardiovascular Outcomes.

**Table 2:** Recent Randomized Controlled Trials on Cardioprotection During Trastuzumab Therapy

Trial	Trial Design	Trial Intervention	Imaging Method	N	Result of Primary Endpoint	Result of Key Secondary Endpoints
Pharmacologic intervention						
MANTICORE 101-Breast^44^	Randomized Placebo-controlled Double-blind Few were treated with anthracyclines	Bisoprolol/perindopril/placebo	CMR	99	No between-group difference in LVEDVi	• Bisoprolol attenuated the decline in LVEF • Perindopril attenuated the decline in LVEF
Boekhout et al^45^	Randomized Multicenter Placebo-controlled Double-blind All were treated with anthracycline in advance	Candesartan/placebo	MUGA	210	No between-group difference in incidence of cardiotoxicity, defined as decline in LVEF of ≥15% or ≤15% to an absolute value < 45%	No between-group differences in changes in LVEF, troponin T, or NT-proBNP
Guglin et al^46^	Randomized Multicenter Placebo-controlled 189 were treated with anthracyclines	Lisinopril/carvedilol/placebo	Echocardiography MUGA	468	No between-group difference in incidence of cardiotoxicity, defined as a reduction in LVEF of ≥10% or a decrease of ≥5% to a value <50%	• Reduction in the incidence of cardiotoxicity if patients treated with sequential anthracyclines in both lisinopril and carvedilol arms • No between-group difference if no anthracycline exposure

LVEDVi = left ventricular end-diastolic indexed volume; MANTICORE 101-Breast = Multidisciplinary Approach to Novel Therapies in Cardio-Oncology Research; MUGA = multigated acquisition; NT-proBNP = N-terminal pro–B-type natriuretic peptide; other abbreviations as in Table 1.

**Table 3:** Ongoing Randomized Trials Evaluating Cardioprotection Strategies

Trial	Trial Number	Cancer	Cancer Therapy	Trial Intervention	Masking/Design	N	Primary Outcome Measures
Pharmacologic intervention: neurohormonal blockade							
PRADA II (Prevention of Cardiac Dysfunction During Breast Cancer Therapy)	NCT03760588	Breast cancer	Anthracyclines with/without trastuzumab/pertuzumab	Sacubitril-valsartan/placebo	Blinded	214	Change in LVEF assessed by CMR from baseline to 18 mo
Carvedilol in Preventing Cardiac Toxicity in Patients With Metastatic HER-2-Positive Breast Cancer	NCT03418961	Metastatic HER2-positive breast cancer	HER2-targeted therapy without concurrent anthracyclines	Carvedilol/no study intervention/observation in patients with increased risk for cardiotoxicity	Single-blinded (outcomes assessor)	817	Time to the first identification of cardiac dysfunction assessed by echocardiography
PROACT (Can We Prevent Chemotherapy-Related Heart Damage in Patients With Breast Cancer and Lymphoma?)	NCT03265574	Breast cancer/lymphoma	Epirubicin	Enalapril/usual care	Single-blinded (outcomes assessor)	170	Cardiac troponin T release during anthracycline treatment (1 mo after last dose of anthracycline)
Effect of Angiotensin Converting Enzyme and Sacubitril Valsartan in Patients After Bone Marrow Transplantation	NCT04092309	Hematological malignancies	Hematopoietic cell transplantation	ACE inhibitor/sacubitril-valsartan/control	Open	90	LVEF by 3D echocardiography/GLS/PWV/glycocalyx thickness
CardioTox (Effects of Carvedilol on Cardiotoxicity in Cancer Patients Submitted to Anthracycline Therapy)	NCT04939883	Cancer patients submitted to anthracycline therapy	Anthracyclines	Carvedilol/placebo	Blinded	1,018	Decline in ejection fraction within 12 mo of starting treatment (>10% to values <50%)/cardiac events
Carvedilol in Preventing Heart Failure in Childhood Cancer Survivors	NCT02717507	Childhood cancer survivors	Anthracyclines	2-y course of low-dose carvedilol/placebo	Blinded	182	LV posterior wall thickness, LV systolic and diastolic function, and afterload; natriuretic peptides, troponins, and galectin-3
Pharmacological interventions: statins							
PREVENT (Preventing Anthracycline Cardiovascular Toxicity With Statins)	NCT01988571	Breast cancer/lymphoma	Anthracyclines	Atorvastatin/placebo	Blinded	279	Change in LVEF by CMR from baseline to 24 mo
STOP-CA (Statins to Prevent the Cardiotoxicity From Anthracyclines)	NCT02943590	Lymphoma	Anthracyclines	Atorvastatin/placebo	Blinded	300	Change in LVEF from baseline to 12 mo assessed by CMR
SPARE-HF (Statins for the Primary Prevention of Heart Failure in Patients Receiving Anthracycline Pilot Study)	NCT03186404	Cancer patients with high CVD risk	Anthracyclines	Atorvastatin/placebo	Blinded	112	Change in LVEF assessed by CMR from baseline to within 4 wk of anthracycline completion
Pharmacological interventions: other							
IPAC (Ivabradine to Prevent Anthracycline-Induced Cardiotoxicity)	NCT03650205	Cancer diagnosis	Anthracyclines	Ivabradine/placebo	Blinded	160	Reduction in GLS of ≥10% from baseline to 12 mo
IPAC (Ivabradine to Prevent Anthracycline-Induced Cardiotoxicity)	NCT04030546	Cancer diagnosis	Anthracyclines	Ivabradine/usual care	Single-blinded (outcomes assessor)	128	Change in GLS at 1, 3, and 6 mo of ≥3%
TRIMETA	EudraCT: 2016-002270-12	HER2-positive breast cancer	Anthracyclines, taxanes, and trastuzumab	Trimetazidine/control	Open	242	Absolute and relative frequency of cardiotoxicity (24 mo) assessed by echocardiography/CREC criteria
Effect of Trimetazidine on Radiotherapy-Induced Heart Damage	NCT04939857	Lung cancer	Stereotactic radiotherapy	Trimetazidine/control	Single-blinded (outcomes assessor)	80	GLS by echocardiography from baseline to 12 mo
Protective Effects of the Nutritional Supplement Sulforaphane on Doxorubicin-Associated Cardiac Dysfunction	NCT03934905	Breast cancer	Doxorubicin	Sulforaphane/placebo	Blinded	70	Change in cardiac function by 2D echocardiography from baseline to 12 mo
Risk/surveillance-guided therapy							
CCT Pilot Guide (Risk-Guided Cardioprotection With Carvedilol in Breast Cancer Patients Treated With Doxorubicin and/or Trastuzumab)	NCT04023110	Breast cancer	Anthracyclines, trastuzumab, or the combination	Risk-guided cardioprotective treatment with carvedilol/usual care	Open	110	Change in LVEF from baseline to 24 mo assessed by echocardiography, treatment adherence, adverse events
COBC (The Cardio-Oncology Breast Cancer Study)	NCT02571894	Breast cancer	Neoadjuvant or adjuvant chemotherapy, with or without trastuzumab	Subclinical cardiotoxicity surveillance and treatment/standard care	Open	320	Event-free survival at 1 y after the completion of chemotherapy
TACTIC (Trastuzumab Cardiomyopathy Therapeutic Intervention With Carvedilol)	NCT03879629	Breast cancer	HER2-targeting therapy	Preemptive vs GLS/troponin guide vs LVEF-guided carvedilol therapy	Open	450	Rate of cardiotoxicity/reversible decline in systolic function assessed by echocardiography from baseline to 12 mo
Strain vs. Left Ventricular Ejection Fraction–Based Cardiotoxicity Prevention in Breast Cancer	NCT04429633	HER2-positive breast cancer	Trastuzumab	Initiation of candesartan guided by decline in LVEF vs GLS	Open	136	Maximum change in LVEF by echocardiography over 18 mo
Cardiac CARE (a randomized trial with breast cancer and lymphoma patients to test if medication can prevent cardiac damage caused by anthracycline chemotherapy)	EudraCT: 2017-000896-99	Breast cancer/lymphoma	Anthracyclines	Troponin-triggered candesartan cilexetil and carvedilol/standard care	Single-blinded (outcomes assessor)	160	Change in LVEF assessed by CMR from baseline to 6 mo after the final anthracycline dose
CARTIER (Cardiovascular Prevention Strategies in Elderly Patients With Cancer)	NCT03711110	Elderly patients with cancer	Standardized antitumor treatment	Intensive cardiovascular monitoring/usual care	Open	514	All-cause mortality: 2 (mid-term analysis) and 5 y of follow-up
TITAN (Multidisciplinary Team Intervention in Cardio-Oncology)	NCT01621659	Breast cancer/lymphoma	Anthracycline and/or trastuzumab-based chemotherapy	Multidisciplinary team intervention/usual care	Single-blinded (outcomes assessor)	80	Change in LVEF assessed by CMR from baseline to 12 mo
NTproBNP-Guide (Pilot Study of an NTproBNP Guided Strategy of Cardioprotection)	NCT04737265	Breast cancer/lymphoma	Anthracyclines	NT-proBNP-guided intervention vs usual care	Open	100	Recruitment, retention, and compliance rate, maximum tolerated dose, incidence of adverse events
SCHOLAR-2 (Safety of Continuing HER-2 Directed Therapy in Overt Left Ventricular Dysfunction)	NCT04680442	HER2-positive breast cancer and evidence of LV dysfunction	Trastuzumab/pertuzumab/trastuzumab-emtansine	Comparing two thresholds of withholding or discontinuing therapy	Blinded	130	Proportion of participants completing trastuzumab/LVEF at the close-out visit and the composite of NYHA functional class III or IV heart failure or cardiovascular death
Exercise							
ATOPE (Attenuating Cancer Treatment-Related Toxicity in Oncology Patients With a Tailored Physical Exercise Program)	NCT03787966	Breast cancer	Surgery, chemotherapy, and radiotherapy	Therapeutic exercise before vs after medical treatment	Single-blinded (outcomes assessor)	110	Change in LVEF by echocardiography from baseline to 12 mo
CAPRICE (Cancer Adverse Effects Prevention With Care & Exercise)	NCT03850171	Breast cancer/lymphoma	Anthracyclines	Exercise training/usual care	Single-blinded (outcomes assessor)	120	Changes in GLS from baseline to 13 wk
ONCORE (Exercise-Based Cardiac Rehabilitation for the Prevention of Breast Cancer Chemotherapy-Induced Cardiotoxicity)	NCT03964142	Breast cancer	Anthracyclines and/or anti-HER2 antibodies	Cardiac rehabilitation program/usual care	Open	122	Change in LVEF and GLS by transthoracic echocardiography during and every year after study completion up to a maximum of 5 y
EXACT2 (Exercise to Prevent Anthracycline-Based Cardio-Toxicity Study 2.0)	NCT03748550	Breast cancer	Anthracyclines	Aerobic exercise/standard care	Single-blinded (outcomes assessor)	100	Change in LVEF from baseline, postintervention (week 13) and 6 mo
Choice of therapy							
RadComp (Pragmatic Randomized Trial of Proton vs Photon Therapy for Patients With Non-Metastatic Breast Cancer: A Radiotherapy Comparative Effectiveness)	NCT02603341	Breast cancer	Radiotherapy	Proton or photon	Open	1,278	Major cardiovascular events at 10 y
RadComp ancillary	NCT04361240	Patients with breast cancer enrolling in the RadComp trial	Radiotherapy	Proton or photon	Open	155	Change in LVEF and RV FAC assessed by echocardiography and NT-proBNP, PIGF, and GDF-15 from baseline to 14 mo
The DBCG Proton Trial: Photon Versus Proton Radiation Therapy for Early Breast Cancer	NCT04291378	Early breast cancer	Radiotherapy	Proton or photon	Open	1,502	Radiation-associated ischemic and valvular heart disease (10 y)
Remote ischemic preconditioning							
ERIC-ONC (Effect of Remote Ischemic Conditioning in Oncology Patients)	NCT02471885	Cancer diagnosis	Anthracyclines	Remote ischemic preconditioning/placebo (sham)	Blinded	128	High-sensitivity troponin T AUC before and after each chemotherapy cycle and at 1-, 3-, 6-, and 12-mo follow-up

Selected ongoing randomized trials of cardioprotection interventions in patients with cancer identified at ClinicalTrials.gov among randomized interventional studies that had not been completed, suspended, terminated, or withdrawn and ClinicalTrialsRegister.eu among randomized trials without results, using the following search terms: “cardiotoxicity,” “cancer and heart failure,” “cancer and cardioprotection,” “cancer and cardiomyopathy,” “cardiovascular toxicity,” and “heart failure and radiotherapy.” In addition, we included selected ongoing trials presented in methods or design papers and recent reviews.

2D = 2-dimensional; ACE = angiotensin-converting enzyme; AUC = area under the curve; CREC = Cardiac Review and Evaluation Committee of trastuzumab-associated cardiotoxicity; CVD = cardiovascular disease; FAC = fractional area change; GDF = growth differentiation factor; HER2 = human epidermal growth factor receptor 2; NYHA = New York Heart Association; PIGF = placental growth factor; PWV = pulse-wave velocity; RV = right ventricular; other abbreviations as in Tables 1 and 2.

**Table 4:** Practical Recommendations for Cardiac Prevention and Treatment Strategies During Anthracycline and/or Trastuzumab Therapy^a^

Identify and treat modifiable cardiovascular risk factors.
In patients with moderate to high cardiovascular risk profile (including but not limited to elevated cardiac troponins and high cumulative anthracycline dose), consider treatment with beta-blockers and/or ACE inhibitors/ARBs.
If cardiac function deteriorates during cancer treatment, suggest treatment with beta-blockers and/or ACE inhibitors/ARBs.
The optimal cardioprotective duration is unknown but should as a minimum be continued during cancer treatment.
If the patient develops signs or symptoms of heart failure, the ability to continue cancer therapy should be discussed with the oncologist/hematologist. Temporary cessation may be necessary, and heart failure treatment should be initiated according to guidelines.
MRAs are considered safe to use.
Sacubitril-valsartan has been associated with beneficial outcomes, but RCTs are lacking.
Reintroduction of cancer therapy under close monitoring and heart failure therapy may be considered after multidisciplinary deliberation depending on cancer type, prognosis, therapy options, severity of cardiotoxicity, and patient preferences.
Optimal treatment duration is unknown.

ACE = angiotensin-converting enzyme; ARB = angiotensin receptor blocker; MRA = mineralocorticoid receptor antagonist; RCT = randomized controlled trials.

a Author group’s recommendations on the basis of expert consensus.

## General Cardioprotective Strategies to Prevent Cancer Therapy Cardiotoxicity

General cardioprotective strategies to prevent cancer therapy cardiotoxicity include both strategies that are common to other forms of cardiovascular disease and some that are specific to cancer and cancer therapy. The multiple-hit model of heart failure is based on the observation that heart failure often is a multifactorial condition.^4^ Extrapolating this theory to the cardiotoxicity setting, patients with established or subclinical cardiovascular disease will have less cardiac functional reserve and therefore tolerate less additional injury before symptoms and signs of cardiotoxicity become clinically apparent. Accordingly, many traditional modifiable and nonmodifiable cardiovascular risk factors are associated with increased risk for CTCRD. Strategies aimed at reducing the risk associated with modifiable risk factors, such as smoking cessation, weight loss, exercise and reduction of sedentary time, and pharmacologic interventions including lipid-lowering, antihypertensive, and antidiabetic therapy, thus have the potential to improve general cardiovascular health status and thereby reduce risk for cardiotoxicity.^5^ These insights provide a strong argument for oncology patients with established cardiovascular disease or those at substantially increased risk for unrecognized subclinical cardiovascular disease to be evaluated by a cardiologist or cardio-oncologist prior to the initiation of potentially cardiotoxic cancer therapy.

Cancer-associated risk markers for cardiotoxicity include the site (eg, pancreatic, kidney, lung, lymphoma) and stage (ie, advanced) of cancer. Cancer associated factors that can increase the risk for cardiovascular disease include hypercoagulability and cancer invasion in the heart and blood vessels, as well as high-output states. Cancer therapy–associated risk factors include prior radiotherapy, especially if directed at the heart and mediastinum, and prior exposure to anthracyclines or hormone therapy.^6^ Although the risk associated with some of these factors is not modifiable, strategies to reduce radiation exposure to the heart and the use of alternative chemotherapeutic agents to anthracyclines are examples of general strategies that have contributed to a lower risk for cardiotoxicity.

## Therapy-Specific Cardioprotective Strategies

### Anthracyclines

#### Oncologic indications and evidence of cardiotoxicity

Anthracyclines are commonly used for the treatment of solid tumors, including breast cancer and sarcomas, as well as hematologic malignancies, and have significantly improved the prognosis of patients with these cancers. Accordingly, withholding anthracyclines may negatively affect cancer outcomes. However, anthracyclines have well-established, dose-dependent, irreversible cardiotoxic effects. The association between cumulative anthracycline dose and risk for heart failure is exponential, with a 5% incidence of heart failure associated with a cumulative dose of 400 mg/m^2^ and a 48% incidence with a cumulative dose of 700 mg/m^2^.^3^ However, the susceptibility to anthracycline-induced cardiotoxicity varies widely according to genetic and cardiovascular risk factors.^7^

#### Cardioprotection against anthracycline-associated cardiotoxicity

##### Measures related to anthracycline administration

Strategies to prevent anthracycline-associated cardiotoxicity include general measures related to anthracycline administration aimed at reducing cytotoxic effects and specific drug interventions aimed at attenuating cardiotoxicity or reducing a deleterious response to injury (Central Illustration, Table 4). General measures related to anthracycline administration include substitution with alternative anticancer drugs, reduction of anthracycline dose, slow infusion rather than bolus injection, and special formulations, such as liposomal doxorubicin. A meta-analysis of randomized controlled trials comparing bolus administration vs continuous infusion and liposomal vs nonliposomal doxorubicin revealed that bolus administration of doxorubicin was associated with a higher rate of clinical and subclinical cardiotoxicity (OR: 4.13; 95% CI: 1.75-9.72), and liposomal formulation was associated with a reduced rate (OR: 0.18; 95% CI: 0.08-0.38).^8^ Moreover, epirubicin was associated with lower risk for clinical cardiotoxicity than doxorubicin (OR: 0.39; 95% CI: 0.20-0.78). Other meta-analyses also are in support of liposomal vs nonliposomal doxorubicin, although the benefit vs epirubicin is less clear.^9^^,^^10^

**Central Illustration:**
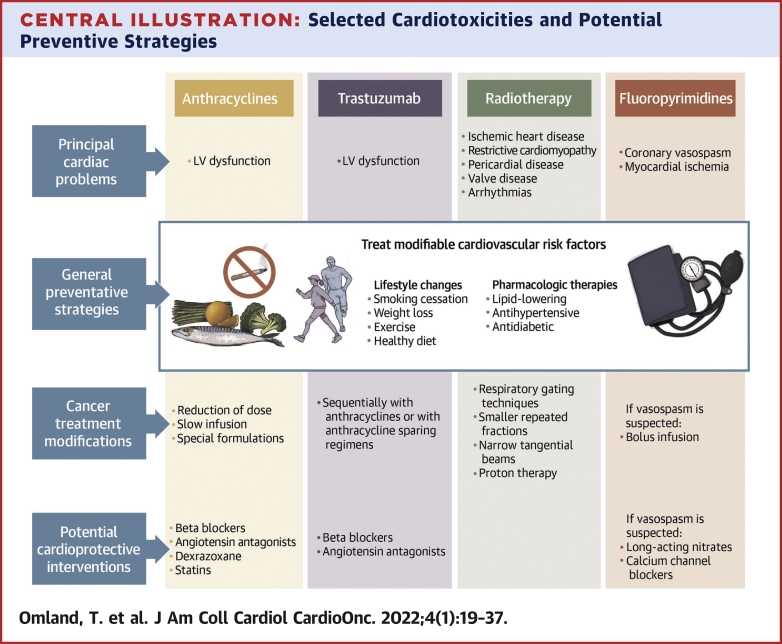
Selected Cardiotoxicities and Potential Preventive Strategies Several classes of anticancer therapies, including anthracyclines, human epidermal growth factor receptor 2 (HER2)–targeted therapy (eg, trastuzumab), radiotherapy, and fluoropyrimidines may cause cardiotoxicity. Whereas the principal cardiotoxic problem associated with anthracyclines and HER2-targeted therapy is left ventricular dysfunction, fluoropyrimidines have been associated with vasospasm and subsequent myocardial ischemia. Radiotherapy may cause a variety of cardiovascular diseases, including ischemic heart disease, valvular and pericardial disease, and cardiomyopathy. Preventive strategies include treatment of modifiable cardiovascular risk factors, modification of cancer treatments, and potentially preventive cardioprotective interventions, but there is need for additional research.

##### Pharmacologic cardioprotective interventions

Specific cardioprotective interventions that have been tested in randomized controlled trials include concurrent treatment with dexrazoxane and concurrent or subsequent treatment with inhibitors of the renin-angiotensin-aldosterone system, beta-adrenoceptor blockers, and statins. Several other potentially protective therapies have also been studied, but the aforementioned interventions have the most robust evidence.^11^

##### Dexrazoxane

Dexrazoxane is an iron-chelating agent with documented cardioprotective effects. Although it was originally thought that the cardioprotective effect of dexrazoxane was related to its iron-chelating properties, leading to cytosolic iron sequestration, more recent evidence suggests that inhibition of doxorubicin-topoisomerase complex formation, leading to reduced apoptosis, ferroptosis, and necroptosis, may also play a role.^12^

Dexrazoxane was approved by the U.S. Food and Drug Administration in 1995 and in 2014 was designated an orphan drug for “prevention of cardiomyopathy for children and adolescents 0 through 16 years of age treated with anthracyclines.”^11^ Concerns related to a potential for reduced anticancer effect and increased risk for secondary malignancies led the European Medicines Agency in 2011 to restrict its use to patients with advanced metastatic breast cancer receiving high cumulative doses of doxorubicin or epirubicin. However, in 2017, the European Medicines Agency overturned its prior decision and allowed dexrazoxane also to be given to children and adolescents who are likely to be treated with high cumulative doses of anthracyclines (>300 mg/m^2^ doxorubicin).

The cardioprotective effect of dexrazoxane has been evaluated in several randomized controlled trials in children, adolescents, and adults. For instance, in the pediatric setting, dexrazoxane administered as a bolus infusion immediately prior to doxorubicin was associated with less reduction in LV fractional shortening and wall thickness than with doxorubicin alone.^13^ An early Cochrane review meta-analysis that included 10 studies of 1,619 patients showed that dexrazoxane was associated with a significant reduction in the pooled estimate of the incidence of heart failure (risk ratio [RR]: 0.29; 95% CI: 0.20-0.41).^14^ In a recent systematic review and meta-analysis that incorporated 2,177 patients from 7 prospective clinical trials and 2 retrospective studies of patients with breast cancer, dexrazoxane significantly reduced the risk for clinical heart failure (RR: 0.19; 95% CI: 0.09-0.40) and cardiac events (RR: 0.36; 95% CI: 0.27-0.49), while the rate of a partial or complete oncological response, overall survival, and progression-free survival appeared to be unaffected in patients with early or metastatic breast cancer receiving anthracyclines with or without trastuzumab.^15^ However, the evidence in early-stage breast cancer is limited, and in the meta-analysis <10% of the cohort had early-stage breast cancer. Notably, none of the included randomized trials were considered to be at low risk for bias across bias domains. Several studies were classified as being at high risk for performance bias because of the unblinded design and attrition bias because of the amount and handling of incomplete outcome data. The investigators appropriately concluded that because of the low quality of the available evidence, further randomized trials are warranted before systematic implementation of dexrazoxane for primary prevention of cardiotoxicity can be recommended in this setting.

Dexrazoxane has also been suggested as a secondary cardioprotective therapy during anthracycline treatment in patients with preexisting ventricular dysfunction. In one consecutive case series, dexrazoxane was used off label concomitantly with anthracyclines. During chemotherapy, mean LV ejection fraction (LVEF) decreased from 39% to 34%, but no patient developed symptomatic heart failure.^16^

##### Neurohormonal blockade strategies

Complex neurohormonal activation may occur as a response to myocardial injury and correlate with the severity of subsequent ventricular dysfunction and heart failure development. These observations form the rationale for neurohormonal antagonists for treatment and prevention of heart failure with beta-adrenergic receptor blockers, angiotensin-converting enzyme (ACE) inhibitors, angiotensin receptor blockers (ARBs), and mineralocorticoid receptor antagonists (MRAs). Intervention with neurohormonal antagonists to attenuate or prevent the deleterious effects of cardiotoxic cancer therapy may therefore seem intuitive.

In 2006, Cardinale et al,^17^ published a seminal paper reporting the results of a randomized, controlled, open-label study of intervention with the ACE inhibitor enalapril initiated 1 month after completion of high-dose chemotherapy and continued for 1 year in a heterogenous cohort of patients with cancer with evidence of acute myocardial injury, reflected in raised cardiac troponin I concentration (>70 ng/L) at the time of high-dose chemotherapy. The effect of enalapril was impressive, with 43% of patients in the control group, but none in the enalapril group, reaching the primary cardiotoxicity outcome of an absolute reduction in LVEF >10% points to a level <50%. The remarkable results of the intervention and the attractive personalized approach to select patients for therapy provided a rationale for further studies with neurohormonal interventions started concomitantly with chemotherapy and for potentially applying this approach to all patients receiving anthracycline therapy.

Following the very promising results of early studies suggesting that intervention with ACE inhibitors and beta-blockers was effective in reversing or attenuating anthracycline-associated cardiotoxicity,^18^^,^^19^ several randomized controlled trials using ACE inhibitors, ARBs, and beta-blockers or their combination were initiated. However, the results of these more recent trials have been mixed, with most reporting modest or no effect of the intervention on the predefined primary outcome measures (Table 1).

Two meta-analyses have recently been published evaluating the results of neurohormonal inhibition in patients receiving anthracyclines and/or trastuzumab.^20^^,^^21^ The larger included 17 trials encompassing 1,984 patients receiving anthracyclines and/or trastuzumab for breast cancer or hematologic malignancies. In pooled analysis, neurohormonal antagonist therapy was associated with higher LVEF on follow-up compared with placebo (mean difference 3.96%; 95% CI: 2.9-5.0). However, because of a high proportion of missing baseline LVEF values, the between-group difference in the change in LVEF was not assessed. Moreover, the incidence of adverse clinical events did not differ significantly between groups (RR: 0.80; 95% CI: 0.53-1.20). Notably, there was significant heterogeneity of the pooled estimates and indications of publication bias, suggesting a need for caution when interpreting the results.^20^

Given the heterogeneity of the studies included in the meta-analyses, some recent randomized controlled cardioprotective trials merit particular discussion. The PRADA (Prevention of Cardiac Dysfunction During Adjuvant Breast Cancer Therapy) trial was a 2 × 2 factorial design trial that randomized 130 patients with early breast cancer to neurohormonal blockade with the ARB candesartan and the beta-blocker metoprolol given concomitantly with anthracycline-containing adjuvant therapy. At the time of completion of blinded therapy (ie, at the time of completion of adjuvant therapy), treatment with candesartan, but not metoprolol, was associated with a statistically significant but numerically modest attenuation in the reduction in LVEF measured by cardiovascular magnetic resonance (CMR), the primary outcome measure, observed in the noncandesartan group (candesartan vs noncandesartan: 0.8% [95% CI: −0.4% to 1.9%] vs 2.6% [95% CI: 1.5% to 3.8%] [*P* = 0.026]; metoprolol vs no metoprolol: 1.6% [95% CI: 0.4% to 2.8%] vs 1.8% [95% CI: 0.7% to 3.0%] [*P* = 0.77]).^22^ When all 4 groups were considered, the decline in LVEF was 2.8% (95% CI: 1.3% to 4.3%) in the placebo-placebo group, 0.9% (95% CI: −0.4% to 2.3%) in the candesartan-placebo group, 2.5% (95% CI: 1.1% to 3.9%) in the metoprolol-placebo group, and 0.6% (95% CI: −0.8% to 2.1%) in the candesartan-metoprolol group. Conversely, treatment with metoprolol was associated with an attenuation of the increase in cardiac troponin I during anthracycline therapy (metoprolol vs no metoprolol: 0.8 ng/L [IQR: 0.8 to 1.2 ng/L] to 4.4 ng/L [IQR: 2.5 to 7.6 ng/L] vs 1.2 ng/L [IQR: 0.8 to 1.5 ng/L] to 7.2 ng/L [IQR: 3.4 to 11.8 ng/L]; between-group difference *P* = 0.019) not observed for candesartan.^23^ Similar results (*P* = 0.020) were observed for cardiac troponin T. Whether the beneficial effects of candesartan and metoprolol were sustained was unclear until extended follow-up data obtained 16 months (IQR: 15 to 19 months) after the completion of blinded therapy were reported.^24^ The extended follow-up data showed no difference in change in LVEF from baseline to extended follow-up between the candesartan vs noncandesartan group or between the metoprolol and nonmetoprolol group (candesartan vs noncandesartan: 1.7% [95% CI: 0.5% to 2.8%] vs 1.8% [95% CI: 0.6% to 3.0%] [between-group difference *P* = 0.91]; metoprolol vs no metoprolol: 1.6% [95% CI: 0.4% to 2.7%] vs 1.9% [95% CI: 0.7% to 3.0%] [between-group difference *P* = 0.73]), or cardiac troponin I (candesartan vs no candesartan: 1.2 ng/L (IQR: −0.6 to 2.9 ng/L) vs 1.9 ng/L [IQR: 0.1 to 3.7 ng/L] [between-group difference *P* = 0.56]; metoprolol vs no metoprolol: 1.4 ng/L [IQR: −0.5 to 3.2 ng/L] vs 1.7 ng/L [95% CI: 0.0 to 3.5 ng/L] [between-group difference *P* = 0.76]).^24^ Accordingly, in this relatively low-risk population, the promising early signals during ongoing neurohormonal blockade did not translate into a sustained beneficial effect on LV systolic function or chronic myocardial injury.

The CECCY (Carvedilol for Prevention of Chemotherapy-Related Cardiotoxicity) trial was a randomized, double-blind, placebo-controlled study of the beta-blocker carvedilol to prevent anthracycline cardiotoxicity in 200 patients with HER2-negative breast cancer.^25^ No between-group difference concerning the primary outcome measure of the incidence of cardiotoxicity, defined as an LVEF reduction of 10% or greater by echocardiography from baseline to 6 months, was observed (carvedilol vs placebo: 14.5% vs 13.5%; *P* = 0.99). Moreover, no between-group difference in the change in LVEF as a continuous variable from baseline to 6 months was observed (*P* = 0.84). However, similar to the observations for metoprolol in PRADA, carvedilol was associated with an attenuation of cardiac troponin I increase during anthracycline therapy (carvedilol vs placebo: 26% vs 41% with values ≥40 ng/L; *P* = 0.003), suggesting a beneficial effect on anthracycline-associated acute myocardial injury. At 2-year follow-up, no differences in the incidence of cardiotoxicity (10% with carvedilol vs 11% with placebo), diastolic dysfunction, change in LVEF, or LV end-diastolic diameter were observed.^26^

Very recently, the preliminary, interim analysis results of a 4-arm, randomized trial (SAFE [Cardiotoxicity Prevention in Breast Cancer Patients Treated With Anthracyclines and/or Trastuzumab]) that evaluated the effect of bisoprolol, ramipril, or their combination to reduce anthracycline-associated subclinical cardiac injury were published as a brief report.^27^ Although the investigators on the ClinicalTrials.gov study site (NCT02236806) state that trial is placebo controlled and double blind and do not specify that interim data will be presented, the published results are based on 12-month follow-up (ie, at end of blinded treatment) with interim data from the first 174 patients included. The interim analysis suggests that the interventions protect against anthracycline-associated decline in LVEF as evaluated using 3-dimensional echocardiography. Accordingly, at 12 months the reduction in LVEF was 4.4% in the placebo group compared with 3.0 %, 1.9%, and 1.3% in the ramipril, bisoprolol, and ramipril plus bisoprolol arms, respectively (*P* = 0.01). Moreover, the coprimary endpoint, worsening of global longitudinal strain (GLS), was 6.0% in the placebo arm and 1.5%, 0.6%, and −0.1% in the ramipril, bisoprolol, and ramipril plus bisoprolol arms, respectively (*P* < 0.001). Although it is uncommon that interim analysis data are published in a double-blind trial if the stopping rule or futility threshold is not reached and the study halted, the primary, end-of-study results at 24 months (ie, 12 months after completion of blinded treatment) of the complete patient sample will provide important information.

The observation that aldosterone is stimulated by angiotensin II and plays an important role in the fibrotic response to myocardial injury provides a rationale for the use of MRAs as a strategy for cardioprotection. So far, sparse and conflicting data exist concerning the effect of MRAs. In a Turkish study, 83 patients with breast cancer receiving anthracycline-containing chemotherapy were randomized to preventive therapy with low-dose spironolactone (25 mg/d) or placebo in a double-blind fashion.^28^ During anthracycline therapy, spironolactone treatment was associated with attenuated deterioration in echocardiographic LVEF (from 67.0% ± 6.1% to 65.7% ± 7.4% in the spironolactone group vs 67.7% ± 6.3% to 53.6% ± 6.8% in the control group; between-group difference *P* < 0.001) and attenuated increase in cardiac troponin I (median 10 to 15 ng/L in the spironolactone group vs 10 to 26 ng/L in the control group; between-group difference *P* = 0.006). However, there was no between-group difference in change in E/e′ ratio (from 8.3 ± 1.6 to 8.5 ± 2.6 in the spironolactone group vs 8.3 ± 2.1 to 9.3 ± 2.8 in the control group; *P* = 0.18). Another study from Canada evaluating the effect of eplerenone on diastolic function (primary endpoint average E′) in patients with breast cancer receiving anthracycline therapy was stopped early because of futility.^29^

The use of combined angiotensin receptor and neprilysin inhibition with sacubitril-valsartan provides more complete neurohormonal inhibition and was associated with reduced mortality and morbidity compared with standard treatment in patients with heart failure with reduced ejection fraction.^30^ The preventive cardioprotective effect of sacubitril-valsartan during (neo)adjuvant therapy in patients scheduled to receive anthracycline-containing therapy is currently being tested in a randomized, placebo-controlled, multicenter trial of patients with early breast cancer (PRADA II [Prevention of Cardiac Dysfunction During Breast Cancer Therapy]; NCT03760588) (Table 3).^31^

##### Statins

In addition to their lipid-lowering effect, statins are known to have pleiotropic anti-inflammatory effects that theoretically may attenuate cancer therapy cardiotoxicity. In a recent meta-analysis, statin therapy was associated with a reduced risk for cardiotoxicity in patients with cancer receiving anthracyclines and/or trastuzumab.^32^ However, most data were derived from observational studies; only 2 small randomized controlled trials with a total of 117 patients were included in the meta-analysis. Although the observational studies suggested significant mitigation of cardiotoxicity after receiving anthracyclines and/or trastuzumab therapy (RR: 0.46; 95% CI: 0.27-0.78; *P* = 0.004), the reduction in risk was not significant in the pooled results of the randomized controlled trials (RR: 0.49; 95% CI: 0.17 to 1.45; *P* = 0.20). Accordingly, the results of the PREVENT (Preventing Anthracycline Cardiotoxicity With Statins; NCT01988571) trial, which has randomized 279 patients with early breast cancer or lymphoma receiving anthracyclines to preventive therapy with atorvastatin vs placebo for 24 months, the STOP-CA (Statins to Prevent the Cardiotoxicity From Anthracyclines; NCT02943590) trial, which has randomized 300 patients with Hodgkin and non-Hodgkin lymphoma receiving doxorubicin to preventive therapy with atorvastatin vs placebo, and SPARE-HF (Statins for the Primary Prevention of Heart Failure in Patients Receiving Anthracyclines Pilot Study; NCT03186404), which aims to randomize 112 patients with cancer scheduled to receive anthracyclines to preventive therapy with atorvastatin vs placebo (Table 3), are eagerly awaited. The primary outcome measures in PREVENT, STOP-CA, and SPARE-HF are change in LV function expressed as LVEF.

##### Risk-guided strategies

A central question concerning cardioprotective therapy to prevent cancer therapy–associated cardiotoxicity is whether the preventive therapy should be administered broadly or in selected groups of patients believed to be at high risk and therefore more likely to benefit from the intervention. Risk-guided strategies rely on the premise that baseline risk or change in risk for cardiotoxicity can be assessed during cancer therapy using imaging markers such as echocardiographic GLS or biochemical markers such as cardiac troponin measured with high-sensitivity assays. A second premise is that the intervention tested has a beneficial effect. Two recent trials have evaluated the effect of a risk-guided strategy, and a third (Cardiac CARE) is ongoing (Table 3). The ICOS-ONE (International CardioOncology Society-one) trial was a randomized, controlled, open-label, multicenter study in which first-in-life patients with cancer from 21 centers in Italy with indications for anthracyclines were assigned to 1 of 2 cardioprotective strategies. One arm of the study started enalapril prior to chemotherapy in all patients, whereas in the other arm enalapril was first given after an abnormal cardiac troponin test result.^33^ The primary outcome was the incidence of cardiac troponin elevation. The study included 273 patients, predominantly women with breast cancer at low cardiovascular risk with a low prevalence of hypertension and diabetes (3% and 4%, respectively). At trial completion, no between-group difference between the 2 approaches was reported for the primary outcome of cardiac troponin elevation (23% in the early prevention vs 26% in the troponin-triggered arm), and the incidence of CTRCD, defined as a reduction in LVEF of 10% or greater to a value <50%, was very low (2 cases in the prevention group, 1 in the troponin-triggered group, 1.1% overall) in both study arms. Moreover, at extended 3-year follow-up, no new cases of incident CTRCD or increased concentrations of cardiac troponin I were reported.^34^ Given the lack of a control group not receiving enalapril, the interpretation of these results remains unclear. Theoretically, both enalapril strategies may have been highly effective in preventing myocardial injury and subsequent CTRCD. Alternatively, and potentially more likely, the results suggest that in a population at low cardiovascular risk receiving contemporary doses of anthracyclines, the risk for sustained myocardial injury and development of CTRCD is modest.

The SUCCOUR (Strain Surveillance of Chemotherapy for Improving Cardiovascular Outcomes) trial was an open, multicenter study randomizing 331 patients receiving anthracycline-containing chemotherapy with 1 or more additional heart failure risk factors to 1 of 2 echocardiographic surveillance strategies for cardiotoxicity using blinded endpoint assessment.^35^

Included patients were assigned to either surveillance with serial measurements of LVEF or with measurement of peak GLS. If incident cardiotoxicity occurred, defined in the standard-of-care arm as an absolute reduction in LVEF of >10% points to <55% or by >5% if accompanied by symptoms, and in the GLS arm as a relative reduction in GLS of ≥12%, therapy with ACE inhibitors or ARBs followed by beta-blockers was initiated. The primary outcome measure in SUCCOUR was the change in LVEF. At 1 year, no significant between-group difference was observed for the primary outcome (−3.0% vs −2.7%; *P* = 0.69) or in secondary outcomes such as change in GLS at 1 year (1.5% vs 1.4%), even though the use of neurohormonal inhibitory therapy was significantly higher in the GLS-guided arm (in 44 of 154 vs 20 of 153 participants). Another secondary endpoint, the incidence of CTRCD, defined as symptomatic LVEF reduction of >5% or >10% asymptomatic to <55%, was lower (5.8 vs 13.7%; *P* = 0.02) in the GLS-guided than in the LVEF-guided trial arm. Although the study design and significance of these results have been debated,^36^^,^^37^ one reasonable interpretation may be that neurohormonal inhibition may be less effective in this setting than previously assumed, as contemporary low to moderate doses of anthracyclines may not induce a strong neurohormonal response. Given that neurohormonal inhibition predominantly modulates the response to injury rather than the cardiotoxic process per se, the effect will be modest in most cases. However, in the absence of a control group not receiving neurohormonal antagonists, the interpretation of the observations of a disconnect between increased use of neurohormonal antagonists and effect on LVEF change remains speculative.

Cardiac CARE (EudraCT 2017-000896-99) is an ongoing, multicenter, prospective, randomized, open-label, endpoint-blinded controlled trial testing a high-sensitivity cardiac troponin–guided combined intervention with the ARB candesartan and the beta-blocker carvedilol to prevent cardiotoxicity in patients with breast cancer and those with lymphoma receiving anthracycline-containing chemotherapy >300 mg/m^2^ (Table 3). The inclusion target is 168 patients, among whom one-third (n = 56) are expected to reach the predefined high-risk cardiac troponin I concentration profile criterion during anthracycline treatment. Patients reaching the high-risk cardiac troponin I criterion will be randomized to candesartan plus carvedilol vs standard care. The primary endpoint will be change in LVEF on CMR from baseline to 6 months after final anthracycline dose in randomized patients. Although sharing some similarities with ICOS-ONE, the use of a standard-of-care arm will provide additional information on the efficacy of combined neurohormonal blockade in high-risk patients.

##### Exercise and lifestyle interventions

Cancer and cancer therapies, in particular anthracyclines, are associated with significant reductions in cardiorespiratory fitness and accelerated physiological aging. Decline in cardiorespiratory fitness in patients receiving cancer therapy should not, however, be ascribed solely to cardiotoxicity but also to the systemic effects of cancer therapy on the skeletal muscle system, which are associated with fatigue and deconditioning. Exercise training and more comprehensive strategies to modify lifestyle may therefore have beneficial effects. In a systematic review and meta-analysis of randomized trials of exercise training in adult patients with cancer, exercise therapy was associated with improved cardiorespiratory fitness (+2.80 mL O_2_ · kg^−1^ · min^−1^ vs 0.02 mL O_2_ · kg^−1^ · min^−1^; *P* < 0.001).^38^ However, randomized controlled trial data specifically evaluating the effect of exercise therapy to prevent or reduce cardiotoxicity are sparse, but this research question will be addressed in ongoing studies (Table 3).

### HER2 Targeted Therapies

#### Oncologic indications and evidence of cardiotoxicity

Trastuzumab is a humanized monoclonal antibody that targets and inhibits HER2. The use of trastuzumab and other monoclonal antibodies directed at HER2, such as pertuzumab, has resulted in markedly improved prognosis for women with HER2-positive breast cancers, both by prolonging survival in advanced, metastatic disease and by reducing the risk for cancer recurrence in the adjuvant setting. Antibody-drug conjugates such as ado-trastuzumab emtansine and trastuzumab-deruxtecan are also used in specific settings for HER2-positive metastatic breast cancer. In addition to breast cancer, antibodies targeting HER2 are also used in HER2-positive gastric and gastroesophageal cancers.

Increased risk for cardiac dysfunction and clinical heart failure in patients receiving trastuzumab was recognized in early trials and occurred most frequently during concurrent anthracycline and trastuzumab treatment. In contrast to anthracycline-associated cardiotoxicity, cardiotoxicity caused by trastuzumab is not associated with cardiomyocyte necrosis histologically, frequently occurs during ongoing therapy, and is commonly considered fully or partly reversible following therapy interruption.^39^

Sequential anthracycline and trastuzumab therapy is associated with increased risk for CTRCD but overall a lower rate of cardiac dysfunction than that observed during concurrent use. In a meta-analysis published in 2011, the incidence was still reported to be relatively high, with asymptomatic declines in systolic function reported to occur in 7.5% and symptomatic heart failure in 2% of patients.^40^ More recent data from clinical trials suggest lower incidence rates. For instance, in the SafeHer (A Safety and Tolerability Study of Assisted and Self-Administered Subcutaneous [SC] Herceptin [Trastuzumab] as Adjuvant Therapy in Early Human Epidermal Growth Factor Receptor 2 (HER2)–Positive Breast Cancer) phase 3 study of subcutaneous trastuzumab for the treatment of HER2-positive early breast cancer, grade ≥3 cardiac disorders were reported in 0.9%, including heart failure in 0.3% of patients, with low event rates both for patients treated with sequential or concurrent chemotherapy.^41^ However, recent population-based studies report higher rates of LV dysfunction than in clinical trials, probably reflecting patients with a higher cardiovascular risk profile. Accordingly, a multicenter cohort study of 10,209 breast cancer survivors demonstrated a 5-year cumulative heart failure incidence of 4.5% among patients treated with sequential anthracycline and trastuzumab therapy, compared with 0.8% in patients treated with anthracyclines only.^42^

Since 2017 the Food and Drug Administration has approved pertuzumab in combination with trastuzumab in adjuvant treatment of HER2-positive breast cancer patients with high risk for recurrence. A higher rate of cardiac dysfunction was anticipated because of the double hit on the HER2 pathway, but major clinical trials suggest that the rate of LV dysfunction is not substantially increased when both drugs are used concomitantly. The cardiotoxicity associated with newer antibody-drug conjugates such as ado-trastuzumab emtansine and trastuzumab-deruxtecan is thought to be lower than for trastuzumab, but data are sparse in patients with previous LV dysfunction.^43^

#### Cardioprotection against trastuzumab-associated cardiotoxicity

##### Pharmacologic cardioprotective interventions

Three recent studies have evaluated the preventive effect of neurohormonal inhibition concomitantly with trastuzumab and provided somewhat diverging results (Table 2). In the MANTICORE 101-Breast (Multidisciplinary Approach to Novel Therapies in Cardio-Oncology Research) trial, 99 patients with HER2-positive early breast cancer, most of whom (77%) had not received prior anthracycline therapy, were randomized in a 1:1:1 double-blind fashion to the beta-blocker bisoprolol, the ACE inhibitor perindopril, or placebo for the duration of trastuzumab therapy.^44^ The trial was stopped early after an interim analysis suggested futility. Accordingly, at the completion of the study, there was no significant difference among groups concerning the primary outcome, LV remodeling expressed as change in indexed LV end-diastolic volume as evaluated by CMR (+7 ± 14 mL/m^2^ with perindopril vs +8 ± 9 mL/m^2^ with bisoprolol vs +4 ± 11 mL/m^2^ with placebo; *P* = 0.36), and MANTICORE 101-Breast should be considered a negative trial. However, in secondary analyses, both perindopril and bisoprolol attenuated the decline in LVEF associated with trastuzumab therapy CMR (−3% ± 4% with perindopril vs −1% ± 5% with bisoprolol vs −5% ± 5% with placebo; *P* = 0.001). Notably, the cardioprotective interventions were well tolerated and associated with fewer interruptions of trastuzumab therapy than placebo.

In a Dutch randomized, multicenter, placebo-controlled, double-blind clinical trial, Boekhout et al^45^ included 210 patients with HER2-positive early breast cancer considered for adjuvant treatment with anthracycline-containing chemotherapy who were randomized to candesartan vs placebo. The predefined primary outcome measure was the incidence of cardiotoxicity, defined as a decline in LVEF, as evaluated by multigated acquisition scanning, of 15% or more or a decrease of <15% to an absolute value <45%. No significant difference in the rate of cardiotoxicity according to this definition was observed 40 weeks after discontinuation of trastuzumab (20 events in the candesartan group vs 16 events in the placebo group, corresponding to 3.8% [95% CI: −7% to 15%; *P* = 0.58] more primary outcome events in the candesartan group). Moreover, candesartan did not affect changes in LVEF, cardiac troponin T, or N-terminal pro–B-type natriuretic peptide as continuous variables compared with placebo.

In a U.S. multicenter, randomized, placebo-controlled, double-blind trial, Guglin et al^46^ included 468 patients with HER2-positive early breast cancer treated with trastuzumab from 127 participating sites who were stratified by prior anthracycline use and assigned in a 1:1:1 fashion to treatment with lisinopril, carvedilol, or placebo. The study intervention started at the beginning of trastuzumab therapy, and follow-up was 12 months after completion of trastuzumab treatment. The primary outcome measure was the incidence of cardiotoxicity, defined as a reduction in LVEF, as evaluated by echocardiography or multigated acquisition scans, of 10% or greater or a decrease of more than 5% if to an absolute value <50%. In the overall cohort, consisting of 189 patients with prior anthracycline exposure and 279 without, there was no significant effect of lisinopril or carvedilol on the primary endpoint (1-tailed *P* values of 0.163 and 0.187 for carvedilol vs placebo and lisinopril vs placebo, respectively). The incidence of cardiotoxicity in this trial was much higher than in most other recent trials, 38% in the stratum with prior anthracycline exposure and 25% in the stratum without, suggesting that potentially this was a higher risk cohort (alternatively, this may be related to study design and outcomes definitions). In stratified analyses, treatment with both lisinopril (HR: 0.53; 95% CI: 0.30-0.94) and carvedilol (HR: 0.49; 95% CI: 0.27-0.89) were associated with a significant reduction in the incidence of cardiotoxicity in the stratum with prior anthracycline exposure. These results seem to favor the theory that the effect of preventive cardioprotective therapy may be greater in high-risk populations.

##### Trastuzumab treatment in patients with preexisting ventricular dysfunction

Even though heart failure has a poor prognosis, a halt in cancer treatment often may portend an even worse prognosis.^47^^,^^48^ Hence, studies on the use of secondary cardioprotective therapy in patients with preexisting, asymptomatic LV dysfunction have been conducted and shown encouraging preliminary results^49^^,^^50^ (Table 5). In SAFE-HEaRt (Cardiac Safety Study in Patients With HER2 + Breast Cancer), 30 women with HER2-positive breast cancer and mildly reduced LVEFs (ie, between 40% and 50%) and no symptoms of heart failure were enrolled. Prior to study start, treatment with beta-blockers and ACE inhibitors was initiated, and during and 6 months after HER2-targeted therapy, patients were carefully monitored with echocardiography and cardiac visits. Treatment was stopped if a cardiac event, defined as heart failure, myocardial infarction, or arrhythmia, occurred or if there was an absolute decline in LVEF of >10% from baseline or LVEF declined to ≤35%. Mean LVEF at baseline was 45% and 46% at the end of treatment. Twenty-seven patients (90%) completed the planned HER2-targeted therapy. Two patients experienced symptomatic heart failure, while 1 had asymptomatic worsening of LVEF to ≤35%.^49^ In SCHOLAR (Safety of Continuing Chemotherapy in Overt Left Ventricular Dysfunction Using Antibodies to HER-2), 20 women with HER2-positive breast cancer, LVEF between 40% and 54%, or a decline in LVEF of ≥15% from baseline were enrolled to examine whether it is safe to continue trastuzumab despite mild cardiotoxicity. Patients received beta-blockers and ACE inhibitors and were followed clinically with echocardiography at a cardio-oncology outpatient clinic. Treatment was stopped if LVEF declined to <40%, accompanied by any heart failure symptoms, or if LVEF declined to <35%. Mean LVEF was 49% at enrollment and 55% at the end of treatment. Eighteen patients (90%) completed the planned HER2-targeted therapy. Two patients developed heart failure with LVEF <40%. Although these results are promising and suggest that in the setting of cardio-oncology care, it may be feasible to continue trastuzumab despite the occurrence of mild cardiotoxicity, larger trials are clearly needed to confirm the safety of HER2-targeted therapy in patients with preexisting mild, asymptomatic ventricular dysfunction.^50^

**Table 5:** Safety Trials for Trastuzumab if Left Ventricular Ejection Fraction Is Reduced

Trial	Trial Inclusion	Trial Intervention	Imaging Method	N	Primary Endpoint	Results
SAFE-HEART^49^	LVEF 40%-49% prior to study participation	Carvedilol and any angiotensin antagonist	Echocardiography	30	Patients completed planned HER2-targeted therapy without developing • Asymptomatic decline in LVEF of >10% from baseline and/or LVEF ≤35% or • Cardiac event, defined as ○ Symptomatic heart failure ○ Cardiac arrhythmia ○ Requiring intervention ○ Myocardial infarction ○ Sudden cardiac death	27 (90%) completed HER2-targeted therapies. 2 developed symptomatic heart failure 1 had asymptomatic LVEF decline to 32%
SCHOLAR^50^	LVEF 40%-54% or LVEF >54% and an absolute fall in LVEF of ≥15% from baseline	Angiotensin-converting enzyme inhibitor and beta-blocker	Echocardiography	20	Cardiac dose-limiting toxicity, defined as • Occurrence of any of the following ○ Cardiovascular death ○ LVEF <40% together with any heart failure symptoms ○ LVEF <35%	2 developed cardiac dose-limiting toxicity

SAFE-HEaRt = Cardiac Safety Study in Patients With HER2 + Breast Cancer; SCHOLAR = Safety of Continuing Chemotherapy in Overt Left Ventricular Dysfunction Using Antibodies to HER-2; other abbreviations as in Tables 1 and 3.

### Cardiac Prevention and Treatment Strategies During Anthracycline and Trastuzumab: Practical Recommendations

Prevention and treatment strategies in the different cardiology and oncology guidelines are to some extent inconsistent. This may be due to slightly differing focus of interest and reflect the time they were written. A summary of practical clinical recommendations is presented in Table 4. In general, an important strategy is to treat modifiable cardiovascular risk factors.^51^ Other preventive measures are modifications of cancer therapy dose and administration method and the administration of potentially cardioprotective drugs such as beta-blockers and or ACE inhibitors or ARBs. The long-term beneficial effects of cardioprotection with these drugs remain unclear, hence a risk-based cardioprotective approach rather than universal implementation may be appropriate at this point.^52^ Accordingly, primary preventive cardioprotective therapy with angiotensin antagonists and/or beta-blockers could be considered in those with moderate to high cardiovascular risk profiles, including elevated cardiac troponin concentrations at baseline or during cancer treatment, and those who receive high cumulative anthracycline doses or display signs of decline in cardiac function.^5^^,^^51^ If symptoms of heart failure develop, cardiac imaging should be performed to assess cardiac function and to determine if cancer treatment should be stopped temporarily and heart failure treatment initiated according to guidelines.^53^ During trastuzumab treatment, it is recommended to halt cancer treatment if LVEF declines to <45% or if the reduction is ≥10% to a value between 45% and 49%.^51^ Before deciding to stop cancer therapy permanently, cardiac imaging should be repeated after 3 weeks to confirm the reduction in LVEF, as there is a significant variability in echocardiographic LVEF measurements.^54^ It is now widely accepted to start cardioprotective treatment with neurohormonal blockade while waiting for a repeat scan, particularly if 3-dimensional LVEF and GLS values also have deteriorated since the last examination. The strongest evidence for dexrazoxane use may be in patients with advanced disease who reach a high cumulative dose of anthracyclines. Definitive data on the efficacy of angiotensin receptor and neprilysin inhibitors, statins, MRAs, and exercise interventions on cardiotoxicity are currently lacking.

### Radiation therapy

Radiotherapy contributes to improved survival rates in a number of thoracic malignancies, such as lymphoma, breast cancer, lung cancer, and esophageal cancer. However, mediastinal radiotherapy may deliver significant radiation doses to the heart and is associated with endocardial, myocardial, and pericardial injury.

Radiotherapy may cause microvascular and macrovascular damage, diffuse interstitial fibrosis, and pericardial and valve disease. Radiotherapy-induced myocardial damage progresses over time, and clinical manifestations include ischemic heart disease due to accelerated coronary artery disease, restrictive cardiomyopathy, heart failure with preserved LVEF, valve regurgitation or stenosis, conduction system injury and arrhythmias, autonomic dysfunction, and pericarditis and pericardial constriction.^55^^,^^56^ In patients with Hodgkin lymphoma, mediastinal radiotherapy was associated with a 2- to 7-fold increase in risk for ischemic heart disease, heart failure, and valvular disease from 10 years after therapy and onward. The risk for radiation-induced heart disease is closely related to cumulative irradiation dose, and a large population-based study of patients with breast cancer demonstrated a linear increase in the rate of major coronary events of 7.4% per Gray mean dose to the heart.^57^ Other identified risk factors include young age, concomitant anthracycline treatment, cardiovascular risk factors, and preexisting cardiovascular disease.^56^, ^57^, ^58^ Different techniques have been introduced to reduce the heart dose during radiotherapy. Conformal and intensity-modulated radiotherapy reduce the dose to organs at risk. Prone positioning and different breathing techniques are used to distance the myocardium from the target volume.^55^^,^^57^, ^58^, ^59^, ^60^ Deep-inspiration breath-hold reduces cardiac radiation dose by administrating radiation when the heart is pulled away from the chest wall during deep breath-holds. In a recent meta-analysis, deep-inspiration breath-hold during radiotherapy for breast cancer was associated with lower radiation dose to the heart (standardized mean difference −1.36; 95% CI: −1.64 to −1.09) and the left anterior descending coronary artery (standardized mean difference −1.45; 95% CI: −1.62 to −1.27).^61^ In a study of 89 patients with left-sided breast cancer, mean heart doses were reduced by 35% (IQR: 23% to 46%) compared with free breathing.^62^ With proton therapy, the finite proton range and increasing dose with depth that peak near the end of range make it possible to adapt the dose distribution and reduce the off-target radiation dose.^63^ The ongoing randomized RadComp (Radiotherapy Comparative Effectiveness) trial will assess the effectiveness of proton vs photon therapy in reducing major cardiovascular events in patients with breast cancer.^64^ However, because of the long latency, radiotherapy-related heart disease from dated treatment regimens is a current issue. In addition, even with contemporary techniques, cardiac irradiation cannot always be avoided, and concerns about radiotherapy-related heart disease remain.

There is a paucity of randomized, controlled trials on the use of cardioprotective medication to prevent radiotherapy-induced myocardial damage in humans, and cardioprotective therapy is not a part of current recommendations.^65^ However, preclinical and observational studies have investigated the potential of statins to decrease the risk for radiotherapy-induced cardiovascular disease.^66^^,^^67^ Colchicine, aspirin, and novel therapies targeting inflammatory pathways may attenuate myocardial inflammation and fibrosis, but clinical evidence of the effect during radiotherapy is lacking.

### Hormone therapy

ADT is the cornerstone of systemic prostate cancer treatment. Patients with prostate cancer are often at elevated risk for cardiovascular disease, as they tend to have a high occurrence of smoking, diabetes, prior myocardial infarction and prior stroke, hypercholesterolemia, hypertension, high body mass index (>30 kg/m^2^), lower muscle strength, and low physical activity.^68^ Additionally, ADT causes changes in risk profile with weight gain, hypertension, and dyslipidemia. Observational studies suggest increased risk for cardiovascular disease, including myocardial infarction, sudden cardiac death, and stroke during ADT treatment.^69^ However, this has not been reproduced in randomized controlled trials.^70^ ADT is commonly given as a gonadotropin-releasing hormone (GnRH) agonist or GnRH antagonist. Observational studies have suggested a stronger relationship with cardiovascular adverse events with GnRH agonists compared with GnRH antagonists.^71^^,^^72^ Meta-analyses confirm the increased risk for cardiovascular disease when comparing GnRH agonist with non-ADT, but this has not been shown for GnRH antagonists.^73^^,^^74^ However, the first international, randomized clinical trial to prospectively compare the cardiovascular safety of a GnRH antagonist with that of a GnRH agonist was published recently and showed no difference between the 2 drugs.^75^ In this study, a total of 545 patients with cardiovascular disease were enrolled, and all patients were seen by a cardiologist. Cardiovascular events were defined as a composite of all-cause death, myocardial infarction, or stroke through 12 months and occurred in 5.5% of patients assigned to the GnRH antagonist compared with 4.1% in in those assigned to GnRH antagonist (HR: 1.28; 95% CI: 0.59-2.79; *P* = 0.53). However, the study was underpowered, as it did not reach its planned inclusion of 900 participants in addition to having fewer than projected primary outcome events.

So far, conventional primary preventive strategies have been suggested and seem reasonable in these patients. Systematic approaches to cardiovascular risk factor modification in these men are being studied.^68^

Two second-generation antiandrogen agents deserve to be mentioned specifically: enzalutamide (an androgen receptor antagonist) and abiraterone (a CYP17 inhibitor). Enzalutamide has in randomized controlled trials been associated with an increased risk for hypertension but not cardiac events.^76^^,^^77^ Abiraterone has been associated with increased risk for both cardiac events and hypertension.^77^^,^^78^

Antiestrogen therapy, including tamoxifen or aromatase inhibitors, may mimic a postmenopausal state but has not been shown to aggravate cardiovascular disease in patients with breast cancer.^79^ Neither tamoxifen nor aromatase inhibitors have been shown to increase the risk for cardiovascular disease in comparison with placebo.^80^ However, tamoxifen has been shown to have a favorable effect on the lipid profile,^80^, ^81^, ^82^ and in a population-based study of 17,922 patients with breast cancer, aromatase inhibitors were associated with increased risks for heart failure and cardiovascular mortality compared with the use of tamoxifen.^83^

### Fluoropyrimidines

Fluoropyrimidines are commonly used for (neo)adjuvant and palliative treatment of colorectal cancer. They can be administrated as bolus (2-15 min), continuous infusion (25-96 h), or orally. Fluoropyrimidines may cause coronary vasospasm resulting in myocardial ischemia with or without electrocardiographic changes.^84^ Symptoms may occur at any time during the treatment period.

Even though randomized placebo-controlled trials are lacking, a commonly accepted strategy to prevent cardiotoxicity from fluoropyrimidines is to optimize modifiable cardiac risk factors. Through case studies it has been shown that reintroduction can be attempted in patients with suspected vasospasm after initiation of long-acting nitrates and/or calcium-channel blockers.^85^^,^^86^ Additionally, bolus injection may be less cardiotoxic, as the vasospasm is thought to be related to accumulated metabolites rather than peak dose. The role of dihydropyrimidine dehydrogenase enzyme deficiency on cardiotoxicity is unclear.

### Other cardiotoxic therapies

There is increasing documentation of a range of cardiovascular toxicities in other commonly used cancer therapeutics, such as immune checkpoint inhibitors, chimeric antigen receptor T-cell therapies, rapidly accelerated fibrosarcoma and mitogen-activated protein kinase kinase inhibitors, proteasome inhibitors, and tyrosine kinase inhibitors. Even though there are some data on how to treat these cardiotoxicities, robust data on primary cardioprotective strategies are lacking.

## Conclusions: Perspectives and Remaining Challenges

Given the incomplete evidence base, there is no clear consensus concerning recommendations for cardioprotective pharmacotherapy. In contrast, there is broad agreement concerning the importance of rigorous risk factor control and treatment, particularly of hypertension. In that context, interaction and collaboration among oncologists, cardiologists, and cardio-oncologists play a central role.

In the absence of definitive, large-scale clinical outcome studies, the question of in whom preventive cardioprotective therapy treatment with neurohormonal antagonists should be initiated remains controversial. Although risk-based strategies to identify patients who will benefit the most are intuitively attractive, existing randomized studies do not yet support the use of imaging- or biomarker-guided interventions. One potential reason for this may be that the effect of neurohormonal antagonist interventions may be relatively minor in the absence of a marked neurohormonal activation. Moreover, neurohormonal antagonists are generally not directed specifically at the cardiotoxic effect of cancer therapies but rather at attenuating the harmful effect of the activation of neurohormonal systems that may occur as a response to myocardial injury. A goal for the future should therefore be to identify new targeted cardioprotective agents.

Clear weaknesses of the existing evidence base concerning cardioprotective therapies for cancer therapy cardiotoxicity are the heterogeneity and modest sample size of most trials. Although many small inconclusive studies should be ideally suited for the conduct of meta-analyses, the considerable heterogeneity in the design, methodology, and patient risk among studies makes the interpretation and generalizability of meta-analyses results challenging. To design larger, collaborative, international multicenter trials should be a high priority to the field of cardio-oncology. The design of conventional clinical trials in oncology has to a large extent been centered exclusively on the efficacy of cancer treatment and has not exploited the potential for obtaining valuable information concerning cardiovascular risk factors and outcomes. To better balance the efficacy of cancer treatment and the risk for cardiotoxicity, collaborative efforts with the pharmaceutical industry and other funders of studies should aim of integrating cardiovascular baseline and outcomes data.

The current use of low to moderate anthracycline doses, the increasing use of non-anthracycline-based chemotherapy alternatives, and enhanced risk factor control have reduced the incidence of CTRCD substantially. Still, the current risk is not negligible and remains high in patient subsets. A continued search for methods to more accurately identify those at increased risk must continue, together with a search for new and more targeted interventions.

## Funding Support and Author Disclosures

Drs Heck and Gulati were supported by grants from the National Programme for Clinical Therapy Research in the Specialist Health Service (KLINBEFORSK). Dr Gulati has received speaker honoraria from Novartis, AstraZeneca, Orion Pharma, and Bristol Myers Squibb. Dr Omland has served on advisory boards for Abbott Diagnostics, Roche Diagnostics, and Bayer; has received research support from Abbott Diagnostics, Novartis, Roche Diagnostics, Singulex, and SomaLogic via Akershus University Hospital; and has received speaker or consulting honoraria from Roche Diagnostics, Siemens Healthineers, and CardiNor. Dr Heck has reported that she has no relationships relevant to the contents of this paper to disclose.

## References

[1] Hahn V.S., Zhang K.W., Sun L., Narayan V., Lenihan D.J., Ky B. (2021). Heart failure with targeted cancer therapies: mechanisms and cardioprotection. Circ Res.

[2] National Cancer Institute NCI Dictionary of Cancer Terms. https://www.cancer.gov/publications/dictionaries/cancer-terms.

[3] Swain S.M., Whaley F.S., Ewer M.S. (2003). Congestive heart failure in patients treated with doxorubicin: a retrospective analysis of three trials. Cancer.

[4] Jones L.W., Haykowsky M.J., Swartz J.J., Douglas P.S., Mackey J.R. (2007). Early breast cancer therapy and cardiovascular injury. J Am Coll Cardiol.

[5] Zamorano J.L., Lancellotti P., Rodriguez Munoz D. (2016). 2016 ESC position paper on cancer treatments and cardiovascular toxicity developed under the auspices of the ESC Committee for Practice Guidelines: the Task Force for Cancer Treatments and Cardiovascular Toxicity of the European Society of Cardiology (ESC). Eur Heart J.

[6] Koene R.J., Prizment A.E., Blaes A., Konety S.H. (2016). Shared risk factors in cardiovascular disease and cancer. Circulation.

[7] Garcia-Pavia P., Kim Y., Restrepo-Cordoba M.A. (2019). Genetic variants associated with cancer therapy-induced cardiomyopathy. Circulation.

[8] Smith L.A., Cornelius V.R., Plummer C.J. (2010). Cardiotoxicity of anthracycline agents for the treatment of cancer: systematic review and meta-analysis of randomised controlled trials. BMC Cancer.

[9] van Dalen E.C., Michiels E.M., Caron H.N., Kremer L.C. (2010). Different anthracycline derivates for reducing cardiotoxicity in cancer patients. Cochrane Database Syst Rev.

[10] Rafiyath S.M., Rasul M., Lee B., Wei G., Lamba G., Liu D. (2012). Comparison of safety and toxicity of liposomal doxorubicin vs. conventional anthracyclines: a meta-analysis. Exp Hematol Oncol.

[11] Bansal N., Adams M.J., Ganatra S. (2019). Strategies to prevent anthracycline-induced cardiotoxicity in cancer survivors. Cardiooncology.

[12] Varghese S.S., Eekhoudt C.R., Jassal D.S. (2021). Mechanisms of anthracycline-mediated cardiotoxicity and preventative strategies in women with breast cancer. Mol Cell Biochem.

[13] Asselin B.L., Devidas M., Chen L. (2016). Cardioprotection and safety of dexrazoxane in patients treated for newly diagnosed T-cell acute lymphoblastic leukemia or advanced-stage lymphoblastic non-Hodgkin Lymphoma: a report of the Children’s Oncology Group Randomized Trial Pediatric Oncology Group 9404. J Clin Oncol.

[14] van Dalen E.C., Caron H.N., Dickinson H.O., Kremer L.C. (2011). Cardioprotective interventions for cancer patients receiving anthracyclines. Cochrane Database Syst Rev.

[15] Macedo A.V.S., Hajjar L.A., Lyon A.R. (2019). Efficacy of dexrazoxane in preventing anthracycline cardiotoxicity in breast cancer. J Am Coll Cardiol CardioOnc.

[16] Ganatra S., Nohria A., Shah S. (2019). Upfront dexrazoxane for the reduction of anthracycline-induced cardiotoxicity in adults with preexisting cardiomyopathy and cancer: a consecutive case series. Cardiooncology.

[17] Cardinale D., Colombo A., Sandri M.T. (2006). Prevention of high-dose chemotherapy-induced cardiotoxicity in high-risk patients by angiotensin-converting enzyme inhibition. Circulation.

[18] Kalay N., Basar E., Ozdogru I. (2006). Protective effects of carvedilol against anthracycline-induced cardiomyopathy. J Am Coll Cardiol.

[19] Bosch X., Rovira M., Sitges M. (2013). Enalapril and carvedilol for preventing chemotherapy-induced left ventricular systolic dysfunction in patients with malignant hemopathies: the OVERCOME trial (Prevention of Left Ventricular Dysfunction With Enalapril and Carvedilol in Patients Submitted to Intensive Chemotherapy for the Treatment of Malignant Hemopathies). J Am Coll Cardiol.

[20] Vaduganathan M., Hirji S.A., Qamar A. (2019). Efficacy of neurohormonal therapies in preventing cardiotoxicity in patients with cancer undergoing chemotherapy. J Am Coll Cardiol CardioOnc.

[21] Lewinter C., Nielsen T., Edfors L. (2021). A systematic review and meta-analysis of beta-blockers and inhibitors of the renin-angiotensin system for preventing left ventricular dysfunction due to anthracyclines or trastuzumab in patients with breast cancer.. Eur Heart J.

[22] Gulati G., Heck S.L., Ree A.H. (2016). Prevention of Cardiac Dysfunction During Adjuvant Breast Cancer Therapy (PRADA): a 2 × 2 factorial, randomized, placebo-controlled, double-blind clinical trial of candesartan and metoprolol. Eur Heart J.

[23] Gulati G., Heck S.L., Rosjo H. (2017). Neurohormonal blockade and circulating cardiovascular biomarkers during anthracycline therapy in breast cancer patients: results from the PRADA (Prevention of Cardiac Dysfunction During Adjuvant Breast Cancer Therapy) study. J Am Heart Assoc.

[24] Heck S.L., Mecinaj A., Ree A.H. (2021). Prevention of Cardiac Dysfunction During Adjuvant Breast Cancer Therapy (PRADA): Extended follow-up of a 2×2 factorial, randomized, placebo-controlled, double-blind clinical trial of candesartan and metoprolol. Circulation.

[25] Avila M.S., Ayub-Ferreira S.M., de Barros Wanderley M.R. (2018). Carvedilol for prevention of chemotherapy-related cardiotoxicity: the CECCY trial. J Am Coll Cardiol.

[26] Ayub-Ferreira S.M., Avila M., Brandao S. (2020). Carvedilol for prevention of chemotherapy-induced cardiotoxicity: final results of the prospective, randomized, double-blind, placebo controlled CECCY trial. J Am Coll Cardiol.

[27] Livi L., Barletta G., Martella F. (2021). Cardioprotective strategy for patients with nonmetastatic breast cancer who are receiving an anthracycline-based chemotherapy: a randomized clinical trial. JAMA Oncol.

[28] Akpek M., Ozdogru I., Sahin O. (2015). Protective effects of spironolactone against anthracycline-induced cardiomyopathy. Eur J Heart Fail.

[29] Davis M.K., Villa D., Tsang T.S.M., Starovoytov A., Gelmon K., Virani S.A. (2019). Effect of eplerenone on diastolic function in women receiving anthracycline-based chemotherapy for breast cancer. J Am Coll Cardiol CardioOnc.

[30] McMurray J.J., Packer M., Desai A.S., PARADIGM-HF Investigators and Committees (2014). Angiotensin-neprilysin inhibition versus enalapril in heart failure.. N Engl J Med..

[31] Mecinaj A., Gulati G., Heck S.L. (2021). Rationale and design of the Prevention of Cardiac Dysfunction During Adjuvant Breast Cancer Therapy (PRADA II) trial: a randomized, placebo-controlled, multicenter trial. Cardiooncology.

[32] Obasi M., Abovich A., Vo J.B. (2021). Statins to mitigate cardiotoxicity in cancer patients treated with anthracyclines and/or trastuzumab: a systematic review and meta-analysis. Cancer Causes Control.

[33] Cardinale D., Ciceri F., Latini R. (2018). Anthracycline-induced cardiotoxicity: a multicenter randomised trial comparing two strategies for guiding prevention with enalapril: the International CardioOncology Society-one trial. Eur J Cancer.

[34] Meessen J., Cardinale D., Ciceri F. (2020). Circulating biomarkers and cardiac function over 3 years after chemotherapy with anthracyclines: the ICOS-ONE trial. ESC Heart Fail.

[35] Thavendiranathan P., Negishi T., Somerset E. (2021). Strain-guided management of potentially cardiotoxic cancer therapy. J Am Coll Cardiol.

[36] Moslehi J.J., Witteles R.M. (2021). Global longitudinal strain in cardio-oncology. J Am Coll Cardiol.

[37] Omland T. (2021). Cardio-protective therapy in cardio-oncology: quo vadis?. Circulation.

[38] Scott J.M., Zabor E.C., Schwitzer E. (2018). Efficacy of exercise therapy on cardiorespiratory fitness in patients with cancer: a systematic review and meta-analysis. J Clin Oncol.

[39] Ewer M.S., Vooletich M.T., Durand J.B. (2005). Reversibility of trastuzumab-related cardiotoxicity: new insights based on clinical course and response to medical treatment. J Clin Oncol.

[40] Chen T., Xu T., Li Y. (2011). Risk of cardiac dysfunction with trastuzumab in breast cancer patients: a meta-analysis. Cancer Treat Rev.

[41] Gligorov J., Ataseven B., Verrill M. (2017). Safety and tolerability of subcutaneous trastuzumab for the adjuvant treatment of human epidermal growth factor receptor 2-positive early breast cancer: SafeHer phase III study’s primary analysis of 2573 patients. Eur J Cancer.

[42] Jacobse J.N., Schaapveld M., Boekel N.B. (2021). Risk of heart failure after systemic treatment for early breast cancer: results of a cohort study. Breast Cancer Res Treat.

[43] Dempsey N., Rosenthal A., Dabas N., Kropotova Y., Lippman M., Bishopric N.H. (2021). Trastuzumab-induced cardiotoxicity: a review of clinical risk factors, pharmacologic prevention, and cardiotoxicity of other HER2-directed therapies. Breast Cancer Res Treat.

[44] Pituskin E., Mackey J.R., Koshman S. (2017). Multidisciplinary Approach to Novel Therapies in Cardio-Oncology Research (MANTICORE 101-Breast): a randomized trial for the prevention of trastuzumab-associated cardiotoxicity. J Clin Oncol.

[45] Boekhout A.H., Gietema J.A., Milojkovic Kerklaan B. (2016). Angiotensin II-receptor inhibition with candesartan to prevent trastuzumab-related cardiotoxic effects in patients with early breast cancer: a randomized clinical trial. JAMA Oncol.

[46] Guglin M., Krischer J., Tamura R. (2019). Randomized trial of lisinopril versus carvedilol to prevent trastuzumab cardiotoxicity in patients with breast cancer. J Am Coll Cardiol.

[47] Chen S.J., Kung P.T., Huang K.H., Wang Y.H., Tsai W.C. (2015). Characteristics of the delayed or refusal therapy in breast cancer patients: a longitudinal population-based study in Taiwan. PLoS ONE.

[48] Mamas M.A., Sperrin M., Watson M.C. (2017). Do patients have worse outcomes in heart failure than in cancer? A primary care-based cohort study with 10-year follow-up in Scotland. Eur J Heart Fail.

[49] Lynce F., Barac A., Geng X. (2019). Prospective evaluation of the cardiac safety of HER2-targeted therapies in patients with HER2-positive breast cancer and compromised heart function: the SAFE-HEaRt study. Breast Cancer Res Treat.

[50] Leong D.P., Cosman T., Alhussein M.M. (2019). Safety of continuing trastuzumab despite mild cardiotoxicity: a phase I trial. J Am Coll Cardiol CardioOnc.

[51] Curigliano G., Lenihan D., Fradley M. (2020). Management of cardiac disease in cancer patients throughout oncological treatment: ESMO consensus recommendations. Ann Oncol.

[52] Gulati G. (2021). Cardioprotection in breast cancer patients—one size fits all?. Eur Heart J.

[53] McDonagh T.A., Metra M., Adamo M. (2021). 2021 ESC guidelines for the diagnosis and treatment of acute and chronic heart failure. Eur Heart J.

[54] Farsalinos K.E., Daraban A.M., Unlu S., Thomas J.D., Badano L.P., Voigt J.U. (2015). Head-to-head comparison of global longitudinal strain measurements among nine different vendors: the EACVI/ASE Inter-Vendor Comparison Study. J Am Soc Echocardiogr.

[55] Ell P., Martin J.M., Cehic D.A., Ngo D.T.M., Sverdlov A.L. (2021). Cardiotoxicity of radiation therapy: mechanisms, management, and mitigation. Curr Treat Options Oncol.

[56] Lancellotti P., Nkomo V.T., Badano L.P. (2013). Expert consensus for multi-modality imaging evaluation of cardiovascular complications of radiotherapy in adults: a report from the European Association of Cardiovascular Imaging and the American Society of Echocardiography. Eur Heart J Cardiovasc Imaging.

[57] Darby S.C., Ewertz M., McGale P. (2013). Risk of ischemic heart disease in women after radiotherapy for breast cancer. N Engl J Med.

[58] Darby S.C., Cutter D.J., Boerma M. (2010). Radiation-related heart disease: current knowledge and future prospects. Int J Radiat Oncol Biol Phys.

[59] Cho B. (2018). Intensity-modulated radiation therapy: a review with a physics perspective. Radiat Oncol J.

[60] Zorn S., Rayan D., Brown S.-A., Bergom C. (2021). Radiation-induced cardiotoxicity. Adv Oncol.

[61] Lai J., Hu S., Luo Y. (2020). Meta-analysis of deep inspiration breath hold (DIBH) versus free breathing (FB) in postoperative radiotherapy for left-side breast cancer. Breast Cancer.

[62] Simonetto C., Eidemuller M., Gaasch A. (2019). Does deep inspiration breath-hold prolong life? Individual risk estimates of ischaemic heart disease after breast cancer radiotherapy. Radiother Oncol.

[63] Paganetti H. (2017).

[64] Bekelman J.E., Lu H., Pugh S. (2019). Pragmatic randomised clinical trial of proton versus photon therapy for patients with non-metastatic breast cancer: the Radiotherapy Comparative Effectiveness (RadComp) Consortium trial protocol. BMJ Open.

[65] Camara Planek M.I., Silver A.J., Volgman A.S., Okwuosa T.M. (2020). Exploratory review of the role of statins, colchicine, and aspirin for the prevention of radiation-associated cardiovascular disease and mortality. J Am Heart Assoc.

[66] Zhang K., He X., Zhou Y. (2015). Atorvastatin ameliorates radiation-induced cardiac fibrosis in rats. Radiat Res.

[67] Boulet J., Pena J., Hulten E.A. (2019). Statin use and risk of vascular events among cancer patients after radiotherapy to the thorax, head, and neck. J Am Heart Assoc.

[68] Leong D.P., Fradet V., Shayegan B. (2020). Cardiovascular risk in men with prostate cancer: insights from the RADICAL PC study. J Urol.

[69] Nguyen-Nielsen M., Moller H., Tjonneland A., Borre M. (2019). Causes of death in men with prostate cancer: results from the Danish Prostate Cancer Registry (DAPROCAdata). Cancer Epidemiol.

[70] O’Farrell S., Garmo H., Holmberg L., Adolfsson J., Stattin P., Van Hemelrijck M. (2015). Risk and timing of cardiovascular disease after androgen-deprivation therapy in men with prostate cancer. J Clin Oncol.

[71] Albertsen P.C., Klotz L., Tombal B., Grady J., Olesen T.K., Nilsson J. (2014). Cardiovascular morbidity associated with gonadotropin releasing hormone agonists and an antagonist. Eur Urol.

[72] George G., Garmo H., Scailteux L.M. (2021). Risk of cardiovascular disease following gonadotropin-releasing hormone agonists vs antagonists in prostate cancer: real-world evidence from five databases. Int J Cancer.

[73] Zhao J., Zhu S., Sun L. (2014). Androgen deprivation therapy for prostate cancer is associated with cardiovascular morbidity and mortality: a meta-analysis of population-based observational studies. PLoS ONE.

[74] Meng F., Zhu S., Zhao J. (2016). Stroke related to androgen deprivation therapy for prostate cancer: a meta-analysis and systematic review. BMC Cancer.

[75] Lopes R.D., Higano C.S., Slovin S.F. (2021). Cardiovascular safety of Degarelix versus leuprolide in patients with prostate cancer: the primary results of the PRONOUNCE randomized trial. Circulation.

[76] Jin C., Fan Y., Meng Y. (2016). A meta-analysis of cardiovascular events in intermittent androgen-deprivation therapy versus continuous androgen-deprivation therapy for prostate cancer patients. Prostate Cancer Prostatic Dis.

[77] Iacovelli R., Ciccarese C., Bria E. (2018). The cardiovascular toxicity of abiraterone and enzalutamide in prostate cancer. Clin Genitourin Cancer.

[78] Moreira R.B., Debiasi M., Francini E. (2017). Differential side effects profile in patients with mCRPC treated with abiraterone or enzalutamide: a meta-analysis of randomized controlled trials. Oncotarget.

[79] Menazza S., Murphy E. (2016). The expanding complexity of estrogen receptor signaling in the cardiovascular system. Circ Res.

[80] Khosrow-Khavar F., Filion K.B., Al-Qurashi S. (2017). Cardiotoxicity of aromatase inhibitors and tamoxifen in postmenopausal women with breast cancer: a systematic review and meta-analysis of randomized controlled trials. Ann Oncol.

[81] Grainger D.J., Schofield P.M. (2005). Tamoxifen for the prevention of myocardial infarction in humans: preclinical and early clinical evidence. Circulation.

[82] Haque R., Shi J., Schottinger J.E. (2016). Cardiovascular disease after aromatase inhibitor use. JAMA Oncol.

[83] Khosrow-Khavar F., Filion K.B., Bouganim N., Suissa S., Azoulay L. (2020). Aromatase inhibitors and the risk of cardiovascular outcomes in women with breast cancer: a population-based cohort study. Circulation.

[84] Polk A., Vaage-Nilsen M., Vistisen K., Nielsen D.L. (2013). Cardiotoxicity in cancer patients treated with 5-fluorouracil or capecitabine: a systematic review of incidence, manifestations and predisposing factors. Cancer Treat Rev.

[85] Clasen S.C., Ky B., O’Quinn R., Giantonio B., Teitelbaum U., Carver J.R. (2017). Fluoropyrimidine-induced cardiac toxicity: challenging the current paradigm. J Gastrointest Oncol.

[86] Padegimas A., Carver J.R. (2020). How to diagnose and manage patients with fluoropyrimidine-induced chest pain. J Am Coll Cardiol CardioOnc.

